# A Stereological Study of the Three Types of Ganglia of Male, Female, and Undifferentiated *Scrobicularia plana* (Bivalvia)

**DOI:** 10.3390/ani12172248

**Published:** 2022-08-31

**Authors:** Sukanlaya Tantiwisawaruji, Maria J. Rocha, Ana Silva, Miguel A. Pardal, Uthaiwan Kovitvadhi, Eduardo Rocha

**Affiliations:** 1 Learning Institute, King Mongkut’s University of Technology Thonburi, Bangkok 10140, Thailand; 2 Laboratory of Histology and Embryology, Department of Microscopy, ICBAS—School of Medicine and Biomedical Sciences, University of Porto (U.Porto), 4050-313 Porto, Portugal; 3 Histomorphology, Physiopathology and Applied Toxicology Group, CIIMAR—Interdisciplinary Centre of Marine and Environmental Research, University of Porto (U.Porto), 4450-208 Matosinhos, Portugal; 4 Centre for Functional Ecology (CFE), Department of Life Sciences, University of Coimbra, 3000-456 Coimbra, Portugal; 5 Department of Zoology, Faculty of Science, Kasetsart University (KU), Bangkok 10900, Thailand

**Keywords:** bivalves, cell numbers, glia cells, ganglia, neurons, sex differences

## Abstract

**Simple Summary:**

In bivalves, neurotransmitters from neural ganglia modulate gonadal maturation, and the gonadal derived sex-steroids may influence the central nervous system. However, it is unknown whether the ganglia microanatomy varies between sexes or with maturation status, besides the intrinsic differences between ganglia types. Therefore, we conducted a quantitative microscopical study on the ganglia of adult peppery furrow shell. Females show a larger glia-to-neuron numerical ratio. Further, females have a greater ganglionic volume than undifferentiated adults, with males showing intermediate values. These facts indicate that ganglia size is related somehow to maturation. Cell size seems to be the basis of the differences. The three ganglion types differ in total volumes and the volume ratio of the cortex versus the medulla. The pedal and visceral ganglia have more voluminous cortexes and medullae, but more neuronal and non-neuronal cells only in the visceral. The mean number of neural cells ranges from 12,000 to over 68,000. The new data make us wonder about the intricate and integrative neural networks of bivalves and how they relate to unsolved issues, such as nociception. The data should help interpret water pollutants’ impacts on the bivalve nervous system, whereas warning that experimental planning should consider sex and gonadal maturation.

**Abstract:**

Neurotransmitters modulate gonadal maturation in bivalves. However, it remains unclear whether there are differences in the nervous system structure between sexes, maturation, and ganglia. Therefore, a stereological study was conducted on the ganglia of adult peppery furrow shell (*Scrobicularia plana*). Equal-sized males, females, and undifferentiated (gamete absence) animals were fixed with 10% formalin and processed for light microscopy. They were serially cut into 35 µm paraffin thick sections and stained with hematoxylin-eosin. Sections with cerebral (cerebropleural), pedal, and visceral ganglia were studied. The parameters estimated were the volumes of the ganglia, the total and relative volumes of their cortex (outer layer) and medulla (neuropil), and the total number of cells (neurons, glia, and pigmented) per ganglia and compartment. The volumes and numbers were estimated, respectively, by the Cavalieri principle and by the optical fractionator. Females show a larger glia to neuron numerical ratio. Further, females have a greater ganglionic volume than undifferentiated adults, with males showing intermediate values. These facts indicate that the ganglia size is related somehow to maturation. The cell size forms the basis of the differences because total cellularity is equal among the groups. The three ganglion types differ in total volumes and the volume ratio of the cortex versus the medulla. The greater volumes of the pedal ganglia (vis-a-vis the cerebral ones) and of the visceral ganglia (in relation to all others) imply more voluminous cortexes and medullae, but more neuronal and non-neuronal cells only in the visceral. The new fundamental data can help interpret bivalve neurophysiology.

## 1. Introduction

Neuroscience studies have shown that in health and disease, the female and male brains of humans and other vertebrates may differ throughout life in various ways [1,2,3]. They also reveal that intrinsic genetic-related neural gene expression, epigenetic influences, and sex-steroidal hormones involved in the regulation of reproduction play central roles in inducing sexually dimorphic brain development and organization [4,5,6]. Moreover, neuronal survival and degeneration are modulated by sex-steroids (neuroprotective), such as estradiol, progesterone, and testosterone [7,8]. These (and other) steroidal hormones are thought to link behaviors either with an internal mechanism, such as ovulation, or with an external factor, such as nutrition [8]. These facts make the brain—and by proxy, the whole central nervous system—a target for the study of central questions as to differences between sexes. For example, it has been asked whether men and women have different brains [9] or whether sex differences in their brain volumes are related to general intelligence [10]. In some vertebrates, neural sexual dimorphism at the structural level has been confirmed. Accordingly, there are articles covering a range of species in which specific zones of the brains were investigated, unveiling differences between males and females regarding the total volume, surface area, cortical thickness, and neuron and glia number [4,11,12,13]. When it was discovered in the human olfactory bulb that females had more neurons and glia, no difference existed in the glia-to-neuron ratio, which is a parameter to watch [12].

In invertebrates, sexual dimorphism in the neural function has been also related to morpho-functional specificities. For instance, fruit flies show signs of sex differences in decision-making behavior in their mating and such modulations seem to occur by the expression of neurons and networks in the fly’s brain [14]. Similar findings regarding olfactory preferences were found in the behavior for the movement and reproduction of nematodes, in which sexual dimorphism is related to specific groups of neurons within a core nervous system shared by both sexes [15]. This specific sensory behavior seems to occur from the functional modulation of common neural circuits controlled by the sex chromosomes [15]. In the malaria mosquito, the reduced acuity of male to odorants was related to dimorphism in the location and number of Orco+ neurons in the antennae and maxillary palps [16].

In bivalves, some examples suggest morphofunctional dimorphism of the nervous system. For instance, in the Pacific lion’s paw scallop, *Nodipecten subnodosis*, the detection of specific monoamines—including dopamine (DA), serotonin (5-HT), and norepinephrine (NA)—was higher in males or females depending on the reproductive cycle periods [17]. The monoamines were found in the gonad, digestive gland, gill, and mantle, where they seem to modulate mechanisms involved in motor behaviors. Most of the 5-HT was registered in the male gonad, at nearly all the maturation stages, except at the spent stage, whereas norepinephrine was abundant in the female gonad [17]. In the visceral ganglia of the New Zealand mussel, *Perna canaliculus*, a few targeted monoamines and neuropeptides were identified by immunohistochemistry and particular expressions were found in small or large neurons of both males and females [18]. Although the latter study did not search for differences between sexes, it unveiled the presence of substances labelled as “responsible for different aspects of reproduction and spawning” and stressed the need to know the influence of seasons and gonad stages on their levels.

From the above, dialogues between the nervous and gonad systems are expected to occur, with the former having major influences on gonadal development and maturation, including in bivalves. Direct and indirect evidence supports the idea for the latter. In the peppery furrow shell, *Scrobicularia plana*, serotonin immunostaining showed stronger intensity and expression extension in the visceral ganglia of mature animals than in immature ones [19]. In the pen shell, *Atrina pectinate*, a neuropepetide detected in visceral ganglia was considered to promote oocyte maturation and sperm motility activation [20]. Despite the neural influences, as seen in other animal taxa, bivalve sex-steroid hormones are primarily present in the gonads [21,22,23]. Despite long-standing controversies about the origin and role of such sex-steroids in bivalves, the endogenous synthesis of estrogens was unequivocally demonstrated recently in the blue mussels, *Mytilus trossulus*, in the gills and gonads of both sexes [24]. Earlier studies had proposed that sex-steroid hormones play a role in the sex differentiation and gonadal development of bivalves, such as the sea scallop, *Placopecten magellanicus* [25]. Despite evidence of the neural and non-neural contributions of peripheral organs to the sexual differentiation of the central nervous system in vertebrates, particularly in humans and rodents [26], there is a knowledge gap in the eventual influences in bivalves. In these, sex and gonadal differentiation is regulated by genetic and epigenetic factors, which do interplay in virtually unknown mechanisms [27,28].

As mentioned, the total volume of the entire nervous system organs and the number of neural cells are parameters found to differ in males and females and vertebrates and invertebrates [12,13,16]. Moreover, those parameters are judged as fundamental quantitative morphological features of interest, to characterize species that may function as experimental models [29], inter alia. Volumes, numbers and other quantities can be accurately and precisely estimated from histological sections by using stereology, a term coined in 1961 when the International Society of Stereology was founded. It is a science based on statistical sampling principles and the stochastic geometric theory. Using specific sampling procedures and measuring and counting protocols, stereology offers unbiased estimates of three-dimensional data (number, length, surface, volume, etc.,) from two-dimensional observations. Stereology solves the biases inherent in simplistic counts and measurements made in microscopy images, the results of which “have remarkably little to with the 3D real world we are interested in” [30]. Stereology has evolved significantly since the late 1980s, with new concepts, procedures, and computer-assisted possibilities.

**Figure 1:**
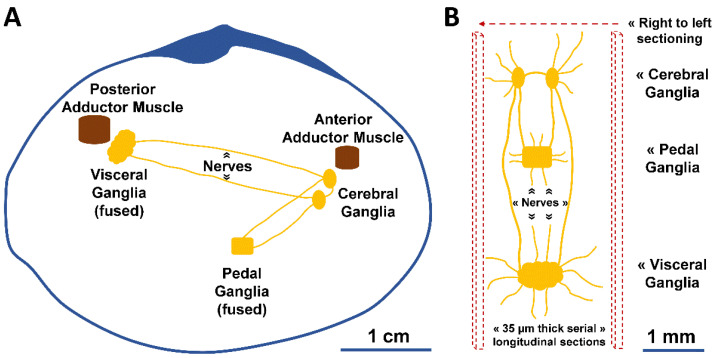
Schematic illustration of the nervous system of *S. plana*. (**A**) Longitudinal view of the right valve, depicting the right and left cerebral (cerebropleural) ganglia, pedal ganglia (fused in one), and visceral ganglia (fused in one). (**B**) Dorsal view of the ganglia, with inter-ganglionic connective nerves and others. The microtome serial sectioning was conducted longitudinally, from right to left.

To quantify neural structures at the microscopical level, from organ size to cell number, the organization of the nervous system and neurocytology of the animal should be known. Here, we used the bivalve peppery furrow shell (*Scrobicularia plana*) as the model organism. The species is of large ecological and economic value [31] and is used as a bioindicator organism for various pollutants [32,33,34,35]. Some disrupt the nervous system, and better descriptions of its structure in bivalves will contribute to better diagnosis, appreciation and prediction of the neurotoxic impacts described in these organisms [36,37,38]. The species’ central nervous system follows the general organization seen in bivalves [19]: three pairs of capsulated ganglia—the right and left cerebral (i.e., cerebropleural) ganglia, the pedal (fused in one) ganglia, and the visceral ganglia (fused in one)—connected by nerves (i.e., nerve cords) (Figure 1). However, the exact location, the relative size of ganglia, and the degree of ganglionic fusion may vary with the bivalve species [39,40].

Considering the above, we hypothesize that because the bivalve ganglia influence the gonadal development and maturation and because the gonadal derived sex-steroids potentially act on the central nervous system, the microscopic morphology of such ganglia, particularly their size and cellularity, may vary between sexes or with maturation status, besides the intrinsic differences that may exist between the different ganglia types. To start tackling these hypotheses, we conducted a stereological study on the nervous ganglia of gonad maturing and of exhausted peppery furrow shell in similarly sized adults, looking into the differences between the sexes and among the three ganglion types. We selected their global and compartmented volumes and cellularity using gold-standard stereology tools [41].

## 2. Materials and Methods

### 2.1. Animals and Histological Procedures

Wild adult peppery furrow shell (*Scrobicularia plana*) were collected at Ria Formosa Lagoon, Portugal. For comparability, the animals were captured on a day in April (Spring) from the same depth and area. They were transferred to in-house facilities on the day of capture and randomly distributed in six glass aquaria (10 L), with aerated seawater (salinity 30‰) at 15 °C. The following day, 6 animals per aquaria were selected, by simple random sampling, for dissection and processing. They were deeply anaesthetized by immersing in a seawater solution of magnesium chloride (6%) and kept at room temperature (≈20 °C). The animal’s length, width, and height were measured with a digital caliper, and the fresh (wet) body and total (wet) mass were evaluated with a digital precision balance.

Each sampled animal was removed carefully from the shell and fixed in toto for 24 h, using 10% neutral phosphate-buffered formalin (pH = 7.2, 0.2 M) at room temperature. The fixed pieces were washed in 70% ethanol, dehydrated with increasing concentrations of alcohol (70–100%), cleared in xylene, and infiltrated with paraffin. Dehydration to infiltration was carried out using an automatic tissue processor (Leica TP1020, Germany). To ensure proper dehydration and infiltration of the animals processed in toto, we used our routine longer protocol, viz., ethanol 70% (1 h), ethanol 90% (24 h), ethanol 96% (48 h), ethanol 99.9% (2 × 48 h), ethanol 99.9%/xylene (72 h), xylene (2 × 24 h), xylene/paraffin (72 h), and paraffin (2 × 48 h). The paraffin blocks were made using an embedding station (Leica EG 1140H, Nussloch, Germany). All the animals were fixed and processed identically for grating comparability.

Each animal was cut into longitudinal (from right to left) serial sections (35 µm in thickness) with a motorized rotary microtome (Leica RM2155, Germany), and kept on 3-aminopropyltriethoxysilane coated slides before hematoxylin and eosin (H&E) staining, xylene clearing, and DPX mounting. The targeted number of animals for the quantitative study was 6 per condition, as defined after histological confirmation, i.e., males, females and undifferentiated (gamete absence). Sections having neural ganglia were used for stereology (others were occasionally used for sexing the animal). The left cerebral, right cerebral, pedal, and visceral ganglia were the targets. Their anatomical positioning in the animal and interconnections by nerves are schematically depicted in Figure 1. General qualitative observations were made with a light microscope (BX50, Olympus, Tokyo, Japan).

### 2.2. Stereological Analysis

Cavalieri’s principle was used for estimating the volume (V) of each ganglion and, separately, of its cortex (outer layer, cell body layer) and medulla (also commonly known as ganglion centrum or neuropil), based on the formula:

V = *t*∙∑ A, (1)

where *t* is the mean distance between the analyzed section planes, and A the sectional area of the target of interest [42]. The raw data estimating the volume of the ganglia were determined semi-automatically using the stereological workstation CAST-Grid (version 1.5, Olympus, Ballerup, Denmark), running with an upright bright field light microscope (BX50, Olympus, Japan), equipped with a microcator with 0.5 µm precision (MT-12, Heidenhain, Traunreut, Germany), a motorized stage with 1 µm X-Y movement accuracy (Prior, Rockland, MA, USA), and a CCD video camera (Sony, Tokyo, Japan) displaying live image in a 17″ CRT monitor (Sony, Tokyo, Japan). Area estimations were done under the ×10 objective lens. For each ganglion, the areas of the cortex and medulla were registered in every serial section across them, to apply later the above-cited formula for V. Areas were automatically computed by the software after the operator manually delineated (using the mouse) the ganglia in the sections. In case a nerve root appeared in the section, it was digitally truncated at its base, so that the nerve would not count for the volume of the respective ganglion body. For each ganglion, *t* was confirmed by measuring the section thickness with the microcator (see below). In practice, the operator manually focused the section top and then moved the focus up to the bottom. The traveling distance *t* was registered by the software. The total ganglionic volumes were used to estimate the relative volumes (V_V_) of the neural cortex and medulla: 10^8^

V_V_ (medulla or cortex, ganglion) = V (medulla or cortex) ÷ V (ganglion). (2)

The total numbers (N) of the neural cells in the ganglia were estimated using the optical disector-fractionator combination [43], adopting the general formula

N = Q ∙ (1 ÷ *ssf*) ∙ (1 ÷ *asf*) ∙ (1 ÷ *hsf*), (3)

where Q refers to the total number of neural cells counted in all the optical disectors; *hsf* is the height sampling fraction, which captures the ratio of the section thickness screened; *asf* is the area sampling fraction, i.e., the ratio between the area of the counting frame and the area covered by each x,y movement (automatically made by the software controlled motorized stage); *ssf* is the section sampling fraction, i.e., the fraction of the total sections sampled. Here, half the total sections of each ganglion were sampled, and a minimum of 100 neurons and 100 glial cells were counted per ganglia. The procedure was enforced semi-automatically with the stereological workstation. The operator’s role was to delineate manually (with the mouse) the limits of the ganglia, insert in the software the x,y movement distances, control the *z* movement in every sampled field, focusing through the disector (in practice a 3D virtual counting box), and count the cells. Counting was done under the ×100 (NA = 1.35) oil immersion lens and in the systematically sampled fields. To check and account for eventual non-uniform deformation, *t* was measured in every field, and as we did not notice such deformation, the averaged *t* was used for *hsf* = *h*/*t* [44]. Here, the average *t* was 33 µm, and the disector *h* was 25 µm. We set a minimal top guard zone of 3 µm, as there is no heterogeneous distribution of cells across the z-axis [45].

As to cellularity, the data are given in various forms, including by splitting the N in numbers per cellular contingents perfectly defined by morphology, as follows: (a) neurons with larger and smaller cell bodies, (b) fusiform, roundish, and triangularly (cell body) shaped glial cells, and (c) pigmented neural cells. These phenotypes were earlier specified in the species [19]. The concept and identification of neurons with larger and smaller somata were absolute because each counted cell body with well-stained cytoplasm was tagged as a “large” (oval/round, often with larger cytoplasmic granules, Ø of ≈ 18 × 25 µm) or as a “small” neuron (oval/round, thick heterochromatin rim, Ø ≈ 7 × 15 µm).

### 2.3. Statistical Analysis

Procedures were done using the software STATISTICA (version 8.0, StatSoft Inc., Tulsa, OK, USA). Data sets were checked for normality and homogeneity of variances—using the Shapiro–Wilk’s W-test and Levene’s test, respectively—before implementing a two-way analysis of variance (two-way ANOVA). After a significant ANOVA, multiple comparisons relied on the Tukey test. At times, logarithmic and square root transformations were carried out for the normalization and homogenization of variances of the raw data. A non-parametric Kruskal–Wallis ANOVA was used when the transformation was unsuccessful, followed by Mann–Whitney U tests for pairs with sequential Bonferroni correction. The significance level was set at 5%. Because a *p*-value does not measure the size of an effect or the importance of a result [46,47], the significance value of *p* ≈ 0.05 was considered a sufficient strength of evidence against the null hypothesis, and therefore a trend toward significance. Data in tables are given as mean (CV–coefficient of variation = standard deviation ÷ mean), giving immediate access to the interindividual variation level, and data in graphs are displayed as mean with a 95% confidence interval.

## 3. Results

### 3.1. Qualitative Histological Observations

**Figure 2:**
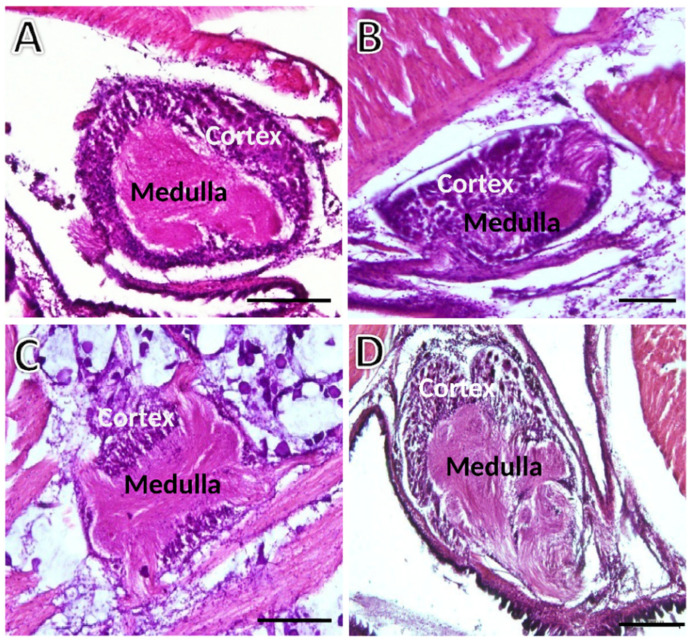
Transversely sectioned ganglia of *S. plana* picked randomly for illustration purposes. (**A**) Left cerebral ganglion. (**B**) Right cerebral ganglion. (**C**) Pedal ganglion. (**D**) Visceral ganglion. The outer basophilic cellular cortex contrasts with the inner medulla. Six animals per sex condition were used. H&E staining. Scale bar = 200 μm.

The general structure of the three ganglion types is presented in Figure 2. The easily distinguishable outer basophilic and highly cellular cortex contrasts with the very eosinophilic inner medulla, essentially composed of neural cell processes.

**Figure 3:**
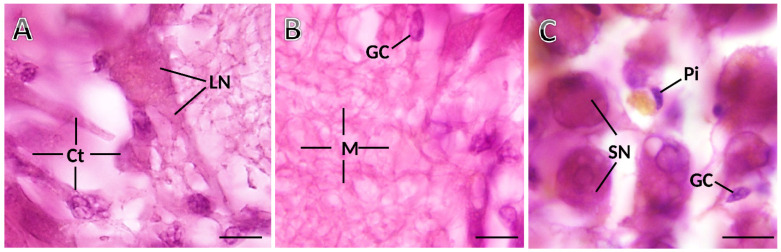
Photomicrographs taken from thick histological sections of a ganglion of *S. plana*. (**A**) Cortex (Ct), with a standing-out large neuron (LN). (**B**) Medulla (M), with neuronal and glial eosinophilic projections, and somata of glial cells (GC). (**C**) Detail of cortex, where smaller neurons (SN), one elongated glial cell (GC) and one pigment cell (Pi) are seen. Six animals per sex condition were used. H&E Staining. Scale bar: 10 μm.

The ganglia have the three previously identified and characterized distinct cell types in *S. plana* [19]: neurons (larger and smaller somata), neuroglia, and pigmented cells (Figure 3). The neurons are unequivocally recognizable by their bigger size, having a single roundish nucleus, typically with one nucleolus, and abundant and well-stained cytoplasm (Figure 3A). The larger neurons are usually located at the outermost cortex, while the smaller ones are aggregated in between the larger and predominating ones in the inner cortex. Neurons and pigmented cells appear in the medulla as well, scarcely but consistently. As for glial cells, they are scattered across the ganglia, amidst neurons and medullar neurites (Figure 3B). The glial cells are distinct from the neurons because the former are much smaller, and have scarce and poorly stained cytoplasm, showing three phenotypes: fusiform, roundish, and triangular. Within each 25 µm-thick optical disector, even roundish glial cells, with their translucent cytoplasm, were easily differentiated from the smaller neurons, because these were evidently more voluminous and had a well-stained (much darker) cytoplasm.

**Figure 4:**
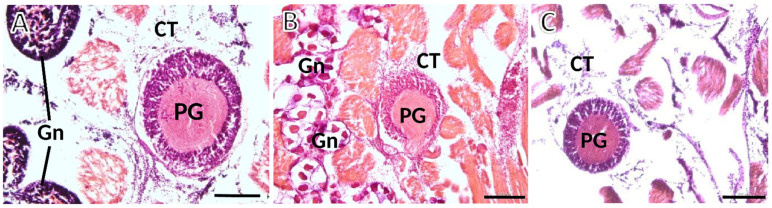
Photomicrographs taken from thick histological sections of ganglia of *S. plana*. (**A**) Male: spermatogenesis is evident within the gonadal acini (Gn); at the upper and lower left corners of the image. (**B**) Female: gonadal acini (Gn) are filled with roundish maturing oocytes. (**C**) Image from one undifferentiated animal, with atrophic acini devoid of maturing gametes, occasionally appearing dispersed in the connective tissue (CT). PG—Pedal ganglion. Six animals per sex were used. H&E staining. Scale bar: 200 μm.

To determine the sex and gonadal status of each animal, we looked at the foot zones near the pedal ganglion. The serial sections enabled precise pinpointing for each specimen which kind of gametes were differentiating, if any, and to classify the animal as male, female, or undifferentiated (Figure 4). In the latter animals, the gonadal acini do not exhibit active gametogenesis, and consequently, sex determination is impossible.

The qualitative observations did not allow the discerning of cellularity differences between animals of different sex conditions (grouped per their gonad differentiation and maturation degree) or across the three ganglia categories—left cerebral (cerebropleural) ganglion (LCG), right cerebral (cerebropleural) ganglion (RCG), pedal ganglion (PG), and visceral ganglion (VG). Hence, we could arrive at conclusions only about obvious features, like the fact that the cortex is more cellular than the medulla and that the visceral ganglion is the biggest of all, though we could not infer how bigger it was, compared to the other types.

### 3.2. Quantitative Data—Body Morphometry

The body biometry of the animals is shown in Table 1. The groups did not statistically differ significantly. The fresh body and the total masses are the most variable parameters.

### 3.3. Quantitative Data—Total and Relative Volumes

**Figure 5:**
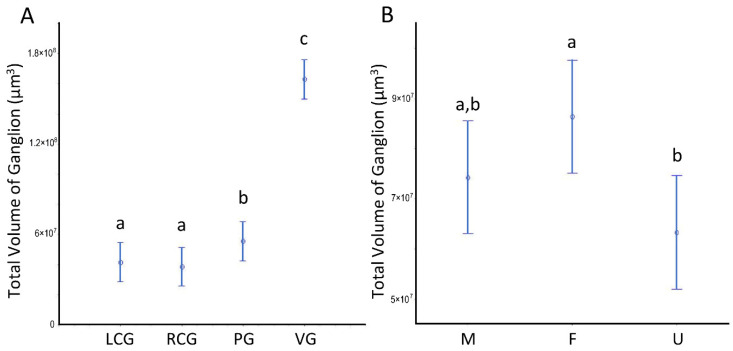
Volumes of the ganglia of *S. plana*. (**A**) Per ganglion. (**B**) All ganglia per sex condition. Different letters mean significant differences (*p* < 0.05). Data as mean and 95% confidence interval. LCG: left cerebral ganglion; RCG: right cerebral ganglion; PG: pedal ganglion; VG: visceral ganglion; M: males; F: females; U: undifferentiated. Six animals per sex condition were used.

The volumes (V) of the three ganglion types are given in Table 2, split by sex condition, and in Figure 5A, with all sexes grouped. The two-way ANOVA highlights a significant effect for the “ganglion type”, where the visceral ganglion V is significantly bigger (3 to 4-fold) than the other ganglia (Figure 5A). Additionally, there is an overall statistical significance as to the parameter sex condition, with females having a greater global/summed ganglionic V than undifferentiated specimens (Figure 5B), while males do not differ from the other groups.

**Figure 6:**
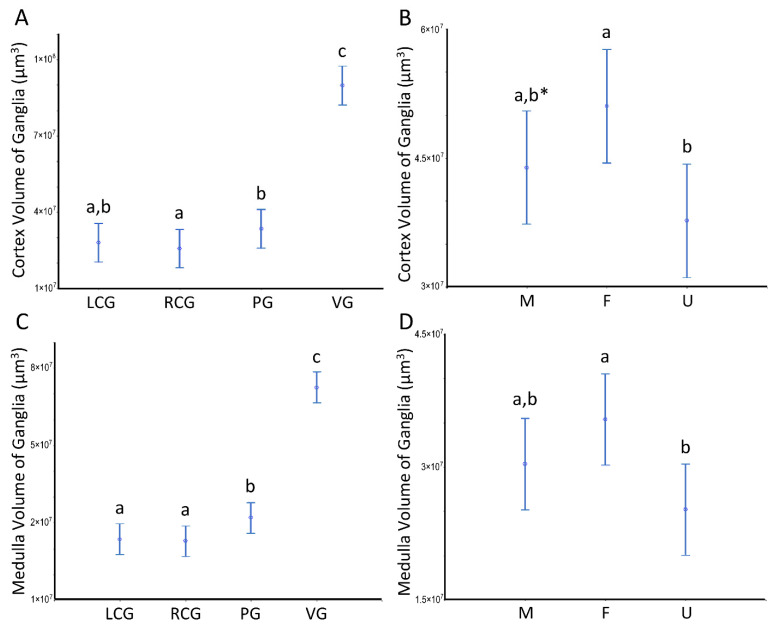
Volumes of the ganglia cortex and medulla of *S. plana*. (**A**) Cortex per ganglion. (**B**) All cortexes per sex condition. (**C**) Medulla per ganglion. (**D**) All medullae per sex condition. Different letters mean significant differences (*p* < 0.05). Data as mean and 95% confidence interval. LCG, RCG, PG, VG, M, F, and U as in Figure 5. * Trend toward a significant difference between M and F (*p* ≈ 0.05). Six animals per sex condition were used.

The volumetric patterns for the ganglia analyzed as a whole also occur when we look at the two key structural ganglionic compartments separately. Indeed, both the cortex and the medulla follow the same trends, as can be seen in Figure 6, with the visceral ganglion compartment standing out from all the others and the females differing from the undifferentiated.

**Figure 7:**
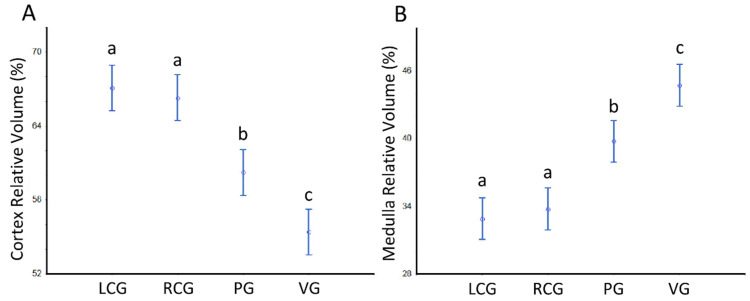
Relative volumes (%) of the cerebral, pedal and visceral ganglia cortex and medulla of *S. plana*, with all sex conditions combined. (**A**) V_V_ (cortex, ganglion). (**B**) V_V_ (medulla, ganglion). Different letters mean significant differences (*p* < 0.05). Data as mean and 95% confidence interval. LCG: left cerebral ganglion; RCG: right cerebral ganglion; PG: pedal ganglia; VG: visceral ganglia. Six animals per sex condition were used.

The V_V_ (cortex, ganglion) and V_V_ (medulla, ganglion) per sex condition are displayed in Table 3 and Table 4. In all ganglion types, the cortex occupies over 50% of the total volume. There are no differences concerning the sex condition effect. However, the analysis reveals a significant effect for the ganglia type (*p* < 0.05), with the V_V_ (cortex, ganglion) decreasing anterior-posteriorly from the cerebral ganglia (that do not differ bilaterally) toward the visceral ganglion; in contrast, the V_V_ (medulla, ganglion) follows a significant reverse pattern, rising toward the visceral. The patterns and the detailed statistical differences between ganglia are displayed in Figure 7.

### 3.4. Quantitative Data—Number of Neural Cells

**Figure 8:**
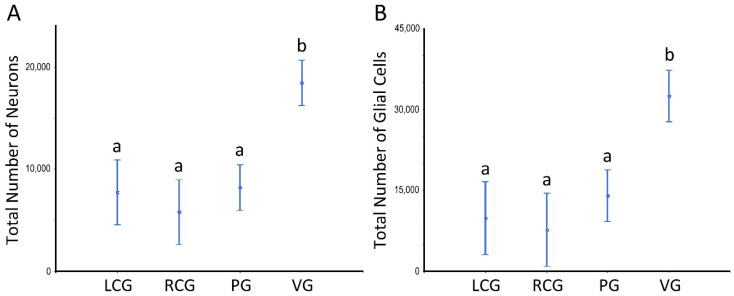
Number of neurons and glial cells in the ganglia of *S. plana*; data from all sex conditions combined. (**A**) Neurons. (**B**) Glial cells. Different letters mean significant differences. Data as mean and 95% confidence interval. LCG: left cerebral ganglion; RCG: right cerebral ganglion; PG: pedal ganglion; VG: visceral ganglion. Six animals per sex condition were used.

**Figure 9:**
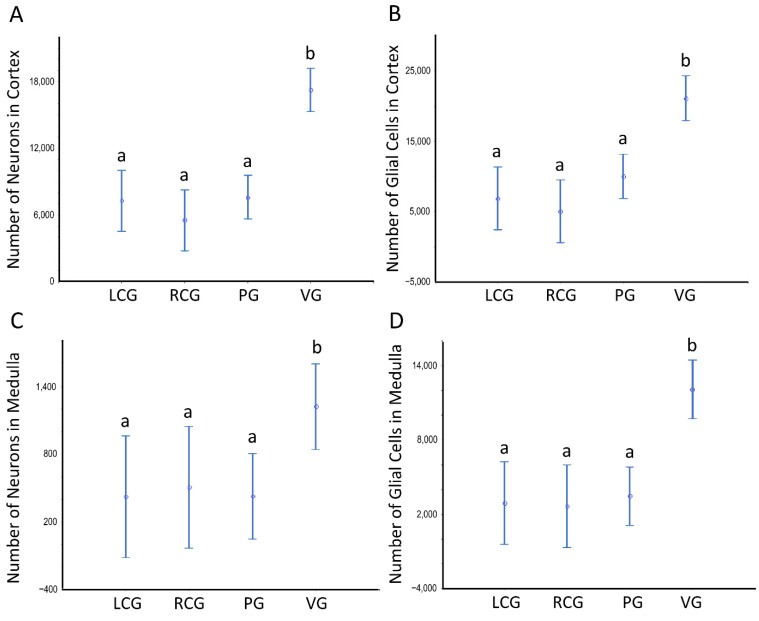
Number of neurons and glial cells in the cortex and medulla of ganglia of *S. plana*; data from all sex conditions combined. (**A**) Neurons in the cortex. (**B**) Glial cells in the cortex. (**C**) Neurons in the medulla. (**D**) Glial cells in the medulla. Different letters mean significant differences. Data as mean and 95% confidence interval (*p* < 0.05). LCG: left cerebral ganglion; RCG: right cerebral ganglion; PG: pedal ganglion; VG: visceral ganglion. Six animals per sex condition were used.

Estimates of the (total) N of neurons (including their partition in the general groups of larger and smaller neurons), of glial cells (sub-divided into fusiform, roundish, and triangular-shaped), and, finally, of pigmented cells data are shown in Figure 8 and Figure 9 and Tables S1–S4; the latter offer data sets for which statistically significant differences exist. The ganglia type and the sex condition are considered in the tabular data, being the sexes grouped in Figure 8 and Figure 9. Table S1 refers to the whole ganglia, while Tables S2 and S3 refer to the cortex and medulla, respectively.

There are no significant differences between sex conditions as to cellularity, despite there being a consistent pattern toward a higher N of pigmented cells in the undifferentiated animals, in parallel to high variability (as denoted by the high CV), which prevents a significant difference. As to the N considering the ganglia type, the ANOVA unveils a significant effect (*p* < 0.001), with the visceral ganglion having statistically significant more neurons and glial cells, but not of pigmented cells. The N of these neural cell types is significantly greater when looking at the whole ganglia (Figure 8) and each of its structural compartments (Figure 9).

Tables S1–S3 illustrate that the subtypes of neurons and glial cells follow patterns similar to those when considering all types. One exception is that the neurons N in the medulla of the visceral ganglion are higher, as in the whole ganglion; but this is so at the cost of the smaller neurons (*p* < 0.05), as the bigger ones do not statistically change. The data shown in Tables S1–S3 translate into numbers, the fact of there being more cells in the cortex, but further demonstrate that the statistic is true for any type of neural cell, not only for neurons. Despite being a minority in the medulla, the N of neurons still reaches from hundreds to ≈ two thousand; as those cells are more erratic in the medulla, the variability associated with the N is higher than in the cortex—as is seen by the relatively higher CV (Table S3) and wider confidence intervals (Figure 9C).

**Figure 10:**
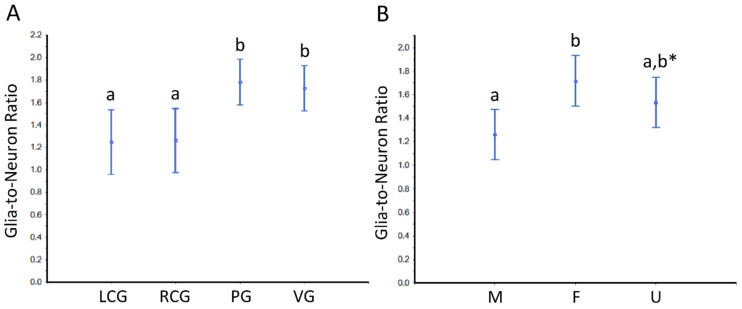
Glia-to-neuron (number) ratio in the ganglia of *S. plana*, considering the whole ganglia. (**A**) Data per ganglion type, irrespective of sex condition. (**B**) Results from all ganglia, grouped per sex condition. Different letters mean significant differences (*p* < 0.05). Data as mean and 95% confidence interval. LCG: left cerebral ganglion; RCG: right cerebral ganglion; PG: pedal ganglion; VG: visceral ganglion; M: males; F: females; U: undifferentiated. * Trend towards a significant difference between M and U (*p* ≈ 0.05). Six animals per sex condition were used.

**Figure 11:**
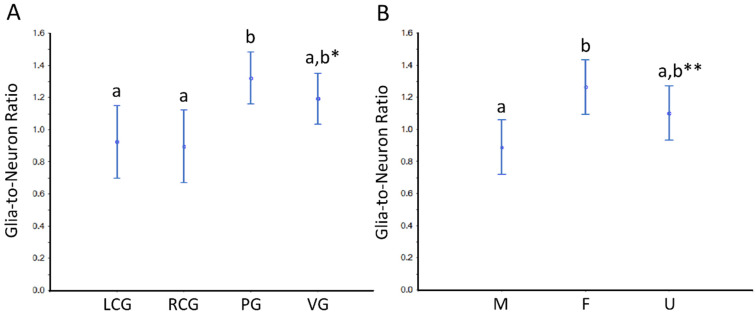
Glia-to-neuron (number) ratio in the cortex of the ganglia of *S. plana*. (**A**) Data per ganglion type, irrespective of sex condition. (**B**) Results from all ganglia, grouped per sex condition. Different letters mean significant differences (*p* < 0.05). Data as mean and 95% confidence interval. LCG: left cerebral ganglion; RCG: right cerebral ganglion; PG: pedal ganglion; VG: visceral ganglion; M: males; F: females; U: undifferentiated. * Trend toward a significant difference between VG and both LCG and RGC (*p* ≈ 0.05). ** Trend towards a significant difference between M and U (*p* ≈ 0.05). Six animals per sex condition were used.

Based on the parameter N, we further estimated the so-called “glia-to-neuron” ratio (Table S4 and Figure 10 and Figure 11). Looking at Table S4, for the whole ganglia, there is a trend for slightly more glial cells than neurons, but if we look at the cortex, the glia-to-neuron ratio is more balanced, at least in the cerebral ganglia. Indeed, the ratio in the cortex is ≈ 1 (LCG ≈ 0.9; RCG ≈ 0.9) and rises in PG ≈ 1.3 and VG ≈ 1.2. In the whole ganglia, thus including medullar neurons and glial cells, the ratio expectably rises in all three ganglia to ≈ 1.5 (LCG ≈ 1.2; RCG ≈ 1.3; PG ≈ 1.7; VG ≈ 1.8). The ratio in the medulla is by nature very high, and as the neurons are erratic, the CVs are higher. The ANOVA revealed no significant interaction between the factors type of ganglia and sex condition, but each factor significantly impacted the ratio (Figure 10 and Figure 11). In conclusion, the glia-to-neuron ratio is higher in the PG and (especially) in the VG, with the females having a higher ratio than males and the undifferentiated being in the middle.

## 4. Discussion

To the best of our knowledge, this is the first study that quantifies bivalve cells and organs with the so-called design-based (i.e., unbiased) stereology. Such designation derives from the fact that its techniques are immune to the biases inherent to several prior generation stereological tools, caused by unrealistic/uncontrolled assumptions about the shape (e.g., assuming perfect sphericity), spatial orientation, and distribution of biological structures in 3D space. The theory and advantages of the design-based methods have been established and refined over the last three decades [41,42,43,44]. However, in bivalves, we find only one article that used such methodology, and not for estimating any component of the animal but rather for revealing the number of the infecting protozoan parasite in the mantle of the eastern oyster *Crassostrea virginica* [48]. The latter and this study illustrate well how the same type of stereological techniques can tackle such varied questions, not to mention their more recently discussed potential to be part of the new field of “morphomics”, in line with the other “omics” [41,49]. The stereology tools used in the present study are well appreciated and recommended in vertebrate neurocytology and have been paramount for sustaining advances [50,51]. Our study is important not only because it unveils new data for *S. plana* that may be used to address hypotheses and support morphofunctional inferences, but also because it uses and details gold-standard stereological procedures, which may stimulate their usage in future bivalve research.

This study aimed to compare the size of the ganglia of *S. plana* (absolute and relative volumes of the whole ganglia and its cortex and medulla) and their cellularity, expressed by the number of neural cells. The theoretical background was a plausible fundamental influence of the gonadal sex in the microanatomy of the bivalve nervous system. We studied males and females, as explicitly identified by their maturing gametes, and specimens that could not be sexed because their gonads were atrophic, at sexual rest. Considering the critical physiological modelling actions of the nervous system on the gametogenesis of bivalves [17,52,53] and the eventual (though not well established yet) feedback loops, we chose to study animals that, at least in theory, should be as functionally dissimilar as *S. plana* adults of different sex could be. We thus opted to analyze three “sex types” to promote the odds of capturing a difference if it existed. This strategy also helped increase the power for studying differences between ganglion types, within the two-way ANOVA, particularly in case of a no significant effect for sex condition—this was relevant here because we wished to strengthen and extend inter-ganglionic differences suggested earlier [54].

As for differences between sex conditions, one that is statistically confirmed concerns the total volume of the ganglia, which is greater in females than in the undifferentiated, with the males not differing from either of the other groups. The cortex and medulla evidence the same type of differences, but there is an additional one in the cortex, with males having a smaller volume (Figure 6B). The insignificant difference between males and females accords with our previous data, which, despite being based on another technique (the much more laborious 3D-reconstruction), offered estimates of the same magnitude and close to those shown here [54]. It would be speculative to assign one particular reason for the difference between females and undifferentiated. However, it is in perfect accordance with our hypothesis that the sex and gonadal status correlate with the bivalve nervous system structure—either because of the activity of the latter in influencing gonads (e.g., [52]) or by the effects of factors originating in the gonad (e.g., sex-steroids) and their impact on the neural elements (e.g., [55]). Whatever the functional implications, our data raises new questions. What causes females to have larger ganglionic volumes than undifferentiated specimens and be slightly larger than males? Are there more neuronal cells or bigger ones? Finally, are there any differences in the amount/size of neural processes? Our data allow us to answer some of these exciting puzzles.

Despite there being no interaction between sex condition and ganglia type, there is a statistically significant effect of the latter in the volumes of the ganglia and their compartments. The two CG are similar in size, but the volumes increase significantly toward the PG, which is greater than the cerebral and much smaller than the VG. The cortical and medullar parts do significantly follow the trends of the whole ganglia. Again, the facts agree with a prior work revealing the same pattern in (adult, but smaller, and thus younger) males and females [54]. We can thus confidently confirm now that the size differences between all ganglion types are independent of the sex condition and the condition of being in the process of gonad maturation.

Besides the absolute volumes, we looked at the relative volumes (V_V_) of the cortex and medulla, quantifying that, overall, the cortex is ≈60% and the medulla 40%. Yet, if the sex condition does not matter for the cortex to medulla ratio, there is a statistically significant effect of the type of ganglia in V_V_, with the less voluminous CG showing the highest mean values for the cortex V_V_, with PG being intermediate, and the VG having the smallest values; conversely, the V_V_ for the medulla followed a matching opposite pattern (Figure 7). This was noted as an approximate trend in a previous study, but was not proven significant [54]. Perhaps such fine structural differences between the V_V_ of the ganglia are not random events and should have a rational and functional impact. One probable reason can be the number of neurites that emerge from the cortex and go into the medulla. In absolute terms, that number is expected to be greater at least in VG, facing the higher total cellularity this ganglion has, compared to the others. Besides the higher number, the size, density and degree of arborization of neuronal and glial projections would promote a relatively greater V_V_ (medulla, ganglion), compared to the other ganglion types. This supposition makes sense because invertebrates have huge plasticity as to the neuropil arborization patterns and synaptic contacts [56]. The lowest cellularity of the LCG and RCG, and consequently fewer projections going into the medullar neuropil, would also explain the smallest V_V_ (medulla, ganglion). On the other hand, such rationale does not explain the intermediate value of the PG, as in this case the total cellularity is no greater than that in either type of CG (see the discussions on cellularity below). Overall, a mixture of morphofunctional factors must be contributing to the differences in V_V_. Among them are the unstudied differences in neuron and glia cell volumes, as well as the degree of complexity in their interconnections, particularly in the cortex of the ganglia, which can have an impact on the volume ratio of cortex-to-medulla. The exact ratio for each ganglion must be linked to the specificities of their functions.

To support the rationale of our discussion, we recall the prior evidence of each ganglia type having specific degrees of organization and function. For instance, the VG is viewed as the most differentiated central nervous system structure in bivalves [57,58]. It has been systematically pointed out that this ganglion is responsible for influencing the cardiac rhythm and motilities of the shell, mantle, siphons, and gills [57,59,60]. As for the PG, it responds to stimulations of the foot with local contractions but requires the cerebral connection to allow digging [57]. The CG play roles in the anterior adductor control, in the coordination of visceral and pedal actions, and are dominant in behavioral rhythms [57]; there can be a dominance of cerebral function [61]. Along with the VG, the CG have roles in respiratory metabolism [62,63]. An overall view of the ganglia functions can be found in [64].

As for neural cellularity, there were no major dissimilarities as to the N of neurons, glial, and pigmented cells, when comparing the LCG and RCG with the PG, despite the latter being significantly bigger. On the other hand, and consistently—i.e., in the whole ganglia and its cortex and medulla—the VG had significantly higher N of neurons and glial cells, but not of pigmented cells. The higher total N in the VG is most likely related to their directly and functionally controlling a vast area of the organism, as stressed earlier [64], which must be based on more neural cells.

When discussing the differences in the absolute volume of ganglia between females and undifferentiated specimens—with females having greater volumes—we questioned what could structurally sustain the dissimilarities. As we did not find differences in the absolute cell numbers, this fact suggests that females must have a higher relative number of cells per unit volume, typically represented in stereology as the N_V_. By dividing the N of cells of a ganglion (or one of its compartments) by its V, we can estimate the N_V_ (cell, containing space). If we investigate this, in the neurons or glia in the cortex, we get an N_V_ in the undifferentiated (of ≈4.3 × 10^5^ neurons/mm^3^ and 5.5 × 10^5^ glia cells/mm^3^) that more than doubles the values of females (of ≈2.0 × 10^5^ neurons/mm^3^ and 2.5 × 10^5^ glia cells/mm^3^), with males situated in between the other sex conditions. When performing this exercise with all neural cells, in all ganglia, we find that the undifferentiated have ≈2.3 more cells per unit of ganglionic volume (≈1.1 × 10^6^ cells/mm^3^), compared either to females (≈4.9 × 10^6^ cells/mm^3^) or males (≈4.7 × 10^6^ cells/mm^3^), that are globally quite similar. These inferences suggest that, overall, the undifferentiated have a similar N of cells in their ganglia fitted into less volume, implying that both the neurons and glial cells are more “concentrated”, and so possibly smaller in size; otherwise, the ganglia V would not be smaller in the undifferentiated. Overall, our data strongly indicate that in *S. plana*, there are sex/gonad stage-related undisclosed differences in the mean volume of neurons and/or glial cells and/or of their projections—the subject of countless studies in vertebrate neuroscience (e.g., [50]) but virtually “untouched” in bivalves. Therefore, the cell sizes are worth studying in the future to understand better the cytology and physiology of the bivalve nervous system.

Still, about cellularity, this is the first study in a bivalve that provides estimates of glia-to-neuron ratios, a fundamental and hotly debated matter in vertebrates, particularly in the human brain, with the once well-established 10:1 ratio later challenged by more rigorous estimates pointing to a 1:1 ratio, supporting the conclusion that humans have an “isometrically scaled-up primate brain” [65,66]. Our global data for *S. plana* (i.e., all ganglia and sex conditions combined) suggest a ≈1:1 glia-to-neuron ratio in the cortex, and when joining the medulla, the ratio rises to ≈1:5. However, the exact ratio depends on sex condition and ganglia type—either factor acting independently—with cerebral ganglia having significantly lower ratios and females showing the highest ratio. Given the neural supportive functions glial cells have across phylogeny, our structural data must have a functional counterpart. Too much speculation should not be made about the new facts, but it is fascinating to note that differences between sexes as to glia-to-neuron ratio were kept along with evolution, up to humans, and that they may also depend on neural regions [12,67]. Our new findings add one more piece to the puzzle of the evolutionary origins of the glia and their continuously gained new roles [68], and offer “ancient roots” in line with the notion that when emerging, brains gained non-neuronal cells in parallel with neuronal additions, resulting in reasonably constant relative densities (i.e., ratios) of non-neuronal cells [69].

## 5. Conclusions

This is the first study to employ design-based stereological methods to accurately estimate the volume of the three anatomical types of ganglia (including their cortex and medulla) as well as the number of neuronal and non-neuronal cells in the entire ganglia and its individual partitions. Females have an overall greater ganglionic volume compared to adults that could not be sexed because they had exhausted gonads. Males exhibited intermediate values. The stereological data supports the notion that ganglia size and gonadal maturation are directly related. The data strongly support the cell size being the basis of the differences in ganglionic volume because there are no significant differences in the total cellularity among the sex conditions studied. Nevertheless, females have a higher glia-to-neuron number ratio than males, with the adult undifferentiated specimens falling somewhere in the middle. This critical cell ratio is significantly the highest in the VG and the lowest in both CG. We further show that the three ganglion types have other fundamental differences, namely in the volume ratio of cortex versus medulla, and that the significantly greater volumes of the PG (compared to the CG) and of the VG (relative to all others) imply more voluminous cortexes and medullae, and more neuronal and non-neuronal cells (only in the VG). We disclose for the first time that such a small bivalve as *S. plana* has a mean total number of neural cells ranging from 12,000 (in CG) to over 68,000 (in VG). Altogether, the new data make us wonder about the intricate and integrative neural networks of bivalves and how they may relate to unsolved mollusc physiology issues, such as nociceptive behavior and their eventual repercussions on animal welfare [70].

## Figures and Tables

**Table 1:** Body morphometry of *S. plana* used in the study.

Sex Condition	Length (cm)	Height (cm)	Width (cm)	Body Mass (g)	Total Mass (g)
Males	3.1 (0.18)	2.5 (0.13)	0.9 (0.12)	1.28 (0.24)	3.03 (0.29)
Females	3.3 (0.06)	2.7 (0.09)	1.1 (0.12)	1.67 (0.22)	4.55 (0.26)
Undifferentiated	3.2 (0.13)	2.4 (0.17)	1.0 (0.21)	1.67 (0.53)	4.57 (0.58)

Six animals per sex condition were used. Data given as mean (coefficient of variation).

**Table 2:** Total volumes (µm^3^) of the cerebral, pedal and visceral ganglia of *S. plana*.

Sex Condition	Cerebral		Pedal	Visceral
	Left	Right		
Males	38.3 × 10^6^ (0.19)	35.6 × 10^6^ (0.23)	59.0 × 10^6^ (0.29)	164.2 × 10^6^ (0.26)
Females	49.8 × 10^6^ (0.23)	48.3 × 10^6^ (0.29)	55.8 × 10^6^ (0.12)	191.7 × 10^6^ (0.32)
Undifferentiated	36.8 × 10^6^ (0.34)	31.9 × 10^6^(0.42)	51.6 × 10^6^ (0.48)	133.0 × 10^6^ (0.32)

Six animals per sex condition were used. Data given as mean (coefficient of variation).

**Table 3:** Relative volumes (%) of the cerebral, pedal, and visceral ganglia cortex of *S. plana*.

Sex Condition	Cerebral		Pedal	Visceral
	Left	Right		
Males	64 (0.02)	63 (0.06)	59 (0.03)	57 (0.12)
Females	68 (0.03)	67 (0.04)	61 (0.09)	54 (0.03)
Undifferentiated	69 (0.04)	68 (0.05)	62 (0.04)	57 (0.09)

Six animals per sex condition were used. Data given as mean (coefficient of variation).

**Table 4:** Relative volumes (%) of the cerebral, pedal and visceral ganglia medulla of *S. plana*.

Sex Condition	Cerebral		Pedal	Visceral
	Left	Right		
Males	36 (0.04)	36 (0.05)	41 (0.05)	43 (0.17)
Females	32 (0.06)	33 (0.08)	39 (0.13)	46 (0.04)
Undifferentiated	31 (0.09)	32 (0.10)	38 (0.06)	43 (0.12)

Six animals per sex condition were used. Data given as mean (coefficient of variation).

## Data Availability

Data are contained within the article or in the supplementary material.

## Supplementary Materials

The following supporting information can be downloaded at: https://www.mdpi.com/article/10.3390/ani12172248/s1. Table S1: Total mean number (N) of neural cells, by ganglia type and sex condition in *S. plana*. Table S2: Total mean number (N) of neural cells in the cortex, by ganglia type and sex condition in *S. plana*. Table S3: Total mean number (N) of neural cells in the medulla, by ganglia type and sex condition in *S. plana*. Table S4: Glia-to-neuron (number) ratio in the cerebral, pedal and visceral ganglia medulla of *S. plana*.
