# Supplementary material for: A Stereological Study of the Three Types of Ganglia of Male, Female, and Undifferentiated Scrobicularia plana (Bivalvia)
Source: Animals (Basel). 2022 Aug 31;12(17):2248. doi: 10.3390/ani12172248 (PMC9454602; doi:10.3390/ani12172248)
Supplement: Supplementary file 1 [file animals-12-02248-s001.zip › animals-1749288-supplementary.pdf]

Supplementary Table S1. Total mean number (N) of neural cells, by ganglia type and sex condition in *S. plana*.

Ganglia	Sex condition	Neurons			Glial cells				Pigmented cells
		Smaller	Larger	Total	Fusiform	Roundish	Triangular	Total	
**LCG**	Males	2,636 (0.3)	4,056 (0.3)	**6,692 (0.3)**	1,613 (0.1)	2,461 (0.4)	1,672 (0.3)	**5,746 (0.2)**	**997 (0.4)**
	Females	2,886 (0.3)	4,977 (0.4)	**7,863 (0.2)**	2,047 (0.6)	3,904 (0.2)	3,584 (0.2)	**9,534 (0.1)**	**1,595 (0.8)**
	Undifferentiated	3,524 (0.4)	5,061 (0.2)	**8,585 (0.3)**	3,088 (0.4)	5,960 (0.6)	4,915 (0.5)	**13,963 (0.5)**	**2,785 (1.3)**
**RCG**	Males	2,430 (0.1)	3,052 (0.3)	**5,482 (0.1)**	1,379 (0.3)	2,416 (0.2)	1,926 (0.4)	**5,721 (0.2)**	**817 (0.9)**
	Females	2,268 (0.1)	3,555 (0.1)	**5,823 (0.1)**	2,866 (0.3)	4,248 (0.6)	2,365 (0.3)	**9,479 (0.3)**	**1,458 (0.4)**
	Undifferentiated	3,149 (0.6)	3,647 (0.5)	**6,796 (0.6)**	2,151 (1.0)	2,697 (0.9)	3,099 (0.2)	**7,947 (0.6)**	**2,785 (1.3)**
**PG**	Males	4,692 (0.4)	3,897 (0.2)	**8,589 (0.2)**	2,871 (0.4)	5,503 (0.4)	3,182 (0.4)	**11,556 (0.3)**	**1,336 (0.5)**
	Females	3,805 (0.2)	3,388 (0.3)	**7,193 (0.2)**	1,682 (0.3)	3,182 (0.5)	7,195 (0.2)	**14,709 (0.3)**	**1,688 (0.5)**
	Undifferentiated	4,745 (0.5)	3,534 (0.5)	**8,279 (0.5)**	2,894 (0.8)	7,438 (0.7)	3,914 (0.3)	**14,246 (0.5)**	**2,906 (0.8)**
**VG**	Males	7,408 (0.2)	8,578 (0.5)	**15,986 (0.3)**	6,200 (0.7)	11,455 (0.4)	8,670 (0.4)	**26,325 (0.4)**	**3,241 (0.8)**
	Females	8,964 (0.1)	9,339 (0.4)	**18,303 (0.3)**	7,833 (0.3)	14,407 (0.2)	10,207 (0.3)	**32,447 (0.2)**	**3,554 (0.6)**
	Undifferentiated	12,437 (0.6)	8,736 (0.4)	**21,173 (0.5)**	7,531 (0.7)	22,138 (0.8)	11,070 (0.4)	**40,739 (0.7)**	**6,251 (1.3)**

Six animals per gender were used. Data given as mean (coefficient of variation). LCG: left cerebral ganglion; RCG: right cerebral ganglion; PG: pedal ganglion; VG: visceral ganglion.

Supplementary Table S2. Total mean number (N) of neural cells in the cortex, by ganglia type and sex condition in *S. plana*.

Ganglia	Sex condition	Neurons			Glial cells				Pigmented cells
		Smaller	Larger	Sum	Fusiform	Roundish	Triangular	Sum	
**LCG**	Males	2310 (0.3)	4001 (0.3)	**6311 (0.3)**	1218 (0.1)	1848 (0.4)	1335 (0.4)	**4401 (0.2)**	**785 (0.4)**
	Females	2745 (0.3)	4798 (0.4)	**7543 (0.2)**	1354 (0.6)	2806 (0.3)	2390 (0.2)	**6550 (0.1)**	**1373 (0.8)**
	Undifferentiated	3125 (0.4)	4887 (0.2)	**8012 (0.3)**	2173 (0.4)	4075 (0.6)	3345 (0.5)	**9593 (0.4)**	**3274 (0.7)**
**RCG**	Males	2171 (0.3)	2975 (0.3)	**5146 (0.2)**	1017 (0.3)	1513 (0.3)	1303 (0.4)	**3833 (0.2)**	**498 (0.7)**
	Females	2059 (0.1)	3273 (0.1)	**5332 (0.1)**	2112 (0.4)	2870 (0.6)	1571 (0.2)	**6554 (0.4)**	**1027 (0.4)**
	Undifferentiated	2801 (0.6)	3296 (0.4)	**6097 (0.5)**	1313 (1.1)	1477 (1.1)	2025 (0.4)	**4815 (0.8)**	**2269 (1.3)**
**PG**	Males	4382 (0.4)	3798 (0.3)	**8180 (0.2)**	2085 (0.4)	4358 (0.5)	2196 (0.4)	**8639 (0.4)**	**978 (0.3)**
	Females	3628 (0.3)	3206 (0.3)	**6834 (0.2)**	2467 (0.6)	5496 (0.2)	3188 (0.4)	**11151 (0.3)**	**1407 (0.6)**
	Undifferentiated	4346 (0.5)	3409 (0.5)	**7755 (0.5)**	2207 (0.8)	5230 (0.8)	2865 (0.4)	**10302 (0.6)**	**2224 (0.9)**
**VG**	Males	6903 (0.2)	8326 (0.5)	**15229 (0.3)**	4362 (0.7)	7279 (0.4)	5091 (0.4)	**16732 (0.5)**	**2178 (0.7)**
	Females	8371 (0.1)	9062 (0.4)	**17433 (0.2)**	5045 (0.3)	9595 (0.2)	6355 (0.3)	**20996 (0.2)**	**2719 (0.5)**
	Undifferentiated	10809 (0.5)	8322 (0.4)	**19131 (0.5)**	5398 (0.7)	12424 (0.6)	7752 (0.4)	**25574 (0.6)**	**5000 (1.2)**

Six animals per gender were used. Data given as mean (coefficient of variation). LCG: left cerebral ganglion; RCG: right cerebral ganglion; PG: pedal ganglion; VG: visceral ganglion.

Supplementary Table S3. Total mean number (N) of neural cells in the medulla, by ganglia type and sex condition in *S. plana*.

Ganglia	Sex condition	Neurons			Glial cells				Pigmented cells
		Smaller	Larger	Sum	Fusiform	Roundish	Triangular	Sum	
**LCG**	Males	325 (0.6)	55 (1.0)	**380 (0.4)**	394 (0.1)	613 (0.5)	337 (0.6)	**1344 (0.2)**	**212 (0.9)**
	Females	141 (1.7)	180 (0.9)	**321 (1.3)**	693 (0.6)	1098 (0.1)	1194 (0.5)	**2985 (0.3)**	**222 (0.7)**
	Undifferentiated	398 (0.5)	174 (1.2)	**572 (0.6)**	916 (0.4)	1885 (0.6)	1569 (0.6)	**4370 (0.5)**	**623 (0.8)**
**RCG**	Males	259 (1.5)	77 (0.9)	**336 (1.3)**	362 (0.5)	903 (0.4)	623 (0.9)	**1888 (0.4)**	**319 (1.3)**
	Females	209 (0.9)	282 (0.5)	**491 (0.5)**	754 (0.2)	1377 (0.6)	794 (0.5)	**2925 (0.2)**	**431 (0.4)**
	Undifferentiated	347 (1.0)	351 (1.5)	**698 (1.2)**	837 (0.8)	1221 (0.6)	1075 (0.3)	**3133 (0.4)**	**516 (1.3)**
**PG**	Males	310 (1.2)	99 (1.3)	**409 (1.2)**	786 (0.6)	1145 (0.6)	986 (0.6)	**2917 (0.4)**	**358 (1.4)**
	Females	178 (1.1)	182 (1.3)	**360 (1.1)**	715 (0.4)	1700 (0.7)	1143 (0.3)	**3558 (0.3)**	**280 (0.3)**
	Undifferentiated	399 (0.7)	126 (1.3)	**515 (0.8)**	687 (0.9)	2208 (0.8)	1049 (0.3)	**3945 (0.6)**	**682 (1.2)**
**VG**	Males	505 (0.7)	252 (1.2)	**757 (0.8)**	1838 (0.7)	4176 (0.3)	3579 (0.4)	**9593 (0.4)**	**1063 (1.1)**
	Females	592 (0.8)	276 (0.8)	**868 (0.6)**	2789 (0.4)	4812 (0.5)	3852 (0.5)	**11452 (0.4)**	**835 (0.9)**
	Undifferentiated	1628 (1.0)	414 (1.0)	**2042 (0.9)**	2133 (0.7)	9714 (1.1)	3318 (0.5)	**15165 (0.8)**	**1251 (1.8)**

Six animals per gender were used. Data given as mean (coefficient of variation). LCG: left cerebral ganglion; RCG: right cerebral ganglion; PG: pedal ganglion; VG: visceral ganglion.

Supplementary Table S4. Glia-to-neuron (number) ratio in the cerebral, pedal and visceral ganglia medulla of *S. plana*.

Ganglia	Sex condition	Glia-to-neuron ratio		
		Whole ganglion	Cortex	Medulla
LCG	Males	0.9 (0.11)	0.7 (0.17)	3.7 (0.24)
	Females	1.3 (0.25)	0.9 (0.25)	26.7 (0.83)
	Undifferentiated	1.3 (0.40)	1.2 (0.23)	8.9 (0.56)
RCG	Males	1.1 (0.14)	0.7 (0.16)	6.9 (0.54)
	Females	1.2 (0.50)	1.2 (0.34)	16.9 (0.98)
	Undifferentiated	1.1 (0.40)	0.7 (0.30)	9.7 (1.13)
PG	Males	1.5 (0.17)	1.0 (0.23)	17.2 (0.91)
	Females	2.2 (0.37)	1.7 (0.21)	18.8 (1.05)
	Undifferentiated	1.7 (0.15)	1.3 (0.14)	6.8 (0.14)
VG	Males	1.6 (0.16)	1.1 (0.26)	13.7 (0.58)
	Females	1.8 (0.13)	1.2 (0.10)	22.6 (0.96)
	Undifferentiated	1.8 (0.22)	1.3 (0.10)	12.1 (1.00)

Six animals per gender were used. Data given as mean (coefficient of variation). LCG: left cerebral ganglion; RCG: right cerebral ganglion; PG: pedal ganglion; VG: visceral ganglion.
